# Lectures on Scarlet Fever

**Published:** 1852-03

**Authors:** 


ARTICLE III.

*Lectures on Scarlet Fever.* By Caspar Morris, M. D., late Lecturer on Practical Medicine, in the Philadelphia Medical Institute, Fellow of the College of Physicians of Philadelphia, Member of the American Philosophical Society, etc. Pages, 104, octavo: Philadelphia; Lindsay & Blakiston, 1851. (From the Publishers through S. C. Griggs & Co., Chicago.

These lectures which originally made their appearance in the Medical Examiner, have been put into the form of a book.

The system of monopaphic treatises is one for which we have before expressed our warmest approbation. We are fully satisfied that it is the only plan upon which a thorough knowledge of the different subjects in our widely extended medical literature can be communicated, and hope that ere long we will have a full library of works, from different authors, each having *investigated thoroughly*, and treated one or two subjects alone. To render such works valuable, would require upon the part of their authors, close and careful investigation, correct and extensive observation, and a candid and sound judgment in reference to both the pathology and treatment of disease.

In the work before us, Dr. Morris has collected much valuable information, both in regard to the history, pathology, and treatment of the disease under consideration. His opposition to active cathartics, and especially to the use of Calomel in Scarlatina, we think is very judicious. We cannot too strongly express our convictions of the deleterious influence of this treatment which we fear is yet too generally resorted to by the profession.

In regard to the new treatment proposed by Dr. Schneeman, the principal feature of which is inunction of the surface by rubbing with a piece of fat bacon, and which has received the sanction of many who have tried it in our country, Dr. Morris speaks very lightly. His principal objection to it, however, it seems to us, will do more to establish his reputation for a fastideous taste, than for a desire to obtain some means of relief from this terrible scourge, the horrors of which he has so forcibly portrayed in the peroration to the subject under discussion.

We quote a paragraph to let our readers understand what we mean:

"Not only is the material used for annointing the skin offensive in itself, but directions are given that the clothing should not be changed, " as a clean shirt takes up more of the fatty matter than one already saturated. The rubbing in is to be kept up twice a day for *three weeks*, and once a day *during the fourth*. The patient is, *after this*, to be washed daily with soap and cool water, and then only is the warm hip bath to be commenced." It would requiie the utmost confidence of success, to reconcile an American mother to such treatment, or induce an American physician to make his daily visits to a chamber so foul. Whatever benefit might accrue from the softening of the cuticle, would be more than balanced by the loathsome effluvia. Nor does the length of time during which the application is to be made, convey an impression at all favorable to the remedial influence of this mode of treatment."

We never understood it to be an essential part of the treatment, that it should be continued so long, nor does the quotation make the continuance of the same linen essential; we therefore ask, if Dr. Morris is not over nice?

We have used the treatment referred to in several cases of Scarlatina, and so greatful has it been to the patients that they have, when old enough, uniformly expressed great satisfaction, and they have so speedily recovered from all distressing symptoms, that it has only been necessary to continue it for a few days.

We always use sudorific anodynes in conjunction with it. Although our observations upon its use in Scarlatina, have not been very extensive, we have formed so favorable an opinion of its influence, that we should not be deterred from again resorting to it, even at the risk of being regarded as indelicate by our author.

The work is got up in the usual good style of the extensive Publishing House, from which it is issued.

